# Hyaluronic acid and chondroitin sulfate in the management of Bacillus Calmette–Guérin–induced cystitis: what we have learned and what is still missing?

**DOI:** 10.3389/fsurg.2026.1783135

**Published:** 2026-03-05

**Authors:** Marilena Gubbiotti, Stefano Rosadi, Valentina Giommoni, Barbara Bigazzi, Emanuele Rubilotta

**Affiliations:** 1 Department of Urology, S. Maria la Gruccia Hospital, Montevarchi, Italy; 2 Department of Urology, AOUI, Verona, Italy

**Keywords:** BCG-induced cystitis, chondroitin sulfate, hyaluronic acid, non-muscle-invasive bladder cancer, urothelial protection

## Introduction

Intravesical Bacillus Calmette–Guérin (BCG) administration is a standardized and widely accepted treatment for high-risk non-muscle-invasive bladder cancer (NMIBC) (1). Despite its well-established oncological efficacy, BCG instillations may induce significant local toxicity, mainly due to damage to the glycosaminoglycan (GAG) layer, which plays a crucial role in maintaining urothelial impermeability against urinary irritants (2). Disruption of this protective barrier, primarily composed of hyaluronic acid (HA), chondroitin sulfate (CS), heparan sulfate, and keratan sulfate, facilitates the penetration of urine constituents, bacteria, ions, and pro-inflammatory mediators into the bladder wall, ultimately triggering a sustained inflammatory response (3). Clinically, this leads to a spectrum of adverse events ranging from mild irritative symptoms to severe chemical cystitis, typically characterized by storage lower urinary symptoms (LUTS), bladder pain, burning, and hematuria (4).

This Opinion Article aims to provide an expert perspective on the pathophysiological rationale and emerging clinical evidence supporting the use of intravesical hyaluronic acid and chondroitin sulfate in the management of BCG-induced chemical cystitis, while highlighting current limitations and unmet needs.

Persistent bladder inflammation may lead to BCG dose reduction, treatment delays, or premature discontinuation, with potential negative impact on oncological outcomes (5). Consequently, the prevention and effective management of BCG-related urinary toxicity should be considered not merely a functional objective but an oncological priority.

Preserving treatment adherence is essential not only to maintain patients’ quality of life (QoL) but also to maximize disease control and survival. Increasing awareness of this issue among clinicians is a fundamental step toward improving daily clinical practice and optimizing BCG delivery. From this perspective, proactive management of BCG-related urinary toxicity should be considered an integral part of the therapeutic pathway rather than a secondary supportive measure. Early interventions aimed at preserving urothelial integrity may not only improve symptom control but also sustain treatment continuity, thereby supporting the overall effectiveness of BCG immunotherapy.

A review of the available literature shows that several strategies have been explored for the management of BCG-related urinary complications. However, similar to radiation-induced chemical cystitis, no optimal or standardized therapeutic approach has yet been clearly established. Intravesical instillation of HA and CS has been proposed for more than a decade as a potential strategy to prevent or mitigate BCG-induced bladder toxicity, and several studies have explored this approach (6). Although the available evidence generally suggests a beneficial effect of HA and CS in reducing urinary symptoms and improving treatment tolerability, the overall impact of these studies is limited by important methodological weaknesses, including small sample sizes, short follow-up periods (often not exceeding 1 year), retrospective or non-randomized design, and heterogeneous treatment protocols (7).

Notably, despite these limitations, a consistent finding across published studies has been a significant reduction in BCG-related side effects and a lower rate of treatment discontinuation, without clinically relevant complications associated with HA and CS instillation (6). However, long-term oncological outcomes have rarely been evaluated, and robust data supporting an indirect benefit on cancer control through improved treatment adherence remain limited.

Recently, a prospective randomized study including more than 100 patients provided stronger evidence supporting the use of highly concentrated HA and CS as add-on therapy during BCG immunotherapy for the management of BCG-induced chemical cystitis (8). In this study, HA and CS were administered after each BCG intravesical instillation according to a simple and reproducible protocol. This approach proved to be effective, safe, and well tolerated, while remaining minimally time-consuming, thus representing a potential foundation for the development of a standardized management strategy. The significant improvement in urinary symptoms and overall treatment tolerability observed in this trial supports the concept that urothelial replenishment therapy may play a pragmatic role in supporting patients undergoing BCG immunotherapy, ultimately enhancing treatment compliance.

These findings have been further reinforced by a recent independent commentary, which underscored the potential clinical value of urothelial replenishment strategies in improving BCG tolerability and adherence (9). Importantly, intravesical GAG therapy demonstrated a favorable safety profile, with no evidence of systemic adverse effects or impairment of BCG efficacy, although additional long-term data are still needed.

In conclusion, evidence accumulated over the last decade suggests that failure to prevent or adequately manage BCG-related urinary toxicity represents both a functional and oncological risk that remains underestimated in routine clinical practice. Effective strategies to reduce BCG-associated complications are available, and an initial standardized protocol supported by prospective randomized data has now been proposed. The next crucial step is to validate these findings through larger, multicenter studies with extended oncological follow-up, which may ultimately lead to a paradigm shift in the supportive management of patients receiving intravesical BCG therapy. In this context, a more proactive and structured approach to preventing BCG-related bladder toxicity appears increasingly justified. Early identification of patients at higher risk of intolerance, combined with the timely implementation of urothelial protective strategies, may represent a key step toward individualized supportive care during BCG immunotherapy. From a broader perspective, integrating symptom control into oncological treatment pathways may help bridge the traditional gap between efficacy and tolerability, ultimately aligning functional preservation with long-term cancer outcomes. This evolving body of evidence supports the concept that optimizing bladder tolerability during BCG therapy should be considered an integral component of high-quality oncological care rather than a merely a supportive intervention.

Further validation through large, multicenter studies using standardized protocols and long-term oncological follow-up is warranted to confirm the clinical and oncological impact of urothelial replenishment strategies during BCG immunotherapy.
